# A Rapid Prototyping Technique for Microfluidics with High Robustness and Flexibility

**DOI:** 10.3390/mi7110201

**Published:** 2016-11-08

**Authors:** Zhenhua Liu, Wenchao Xu, Zining Hou, Zhigang Wu

**Affiliations:** 1 State Key Laboratory of Digital Manufacturing Equipment and Technology, Huazhong University of Science and Technology, Wuhan 430074, China; liuzhenhua@hust.edu.cn (Z.L.); m201470383@hust.edu.cn (W.X.); purewind8@gmail.com (Z.H.); 2 Ångström Laboratory, Microsystems Technology, Department of Engineering Sciences, Uppsala University, Uppsala 75121, Sweden

**Keywords:** prototyping technique, microfluidics, soft lithography, ultraviolet (UV) laser direct writing

## Abstract

In microfluidic device prototyping, master fabrication by traditional photolithography is expensive and time-consuming, especially when the design requires being repeatedly modified to achieve a satisfactory performance. By introducing a high-performance/cost-ratio laser to the traditional soft lithography, this paper describes a flexible and rapid prototyping technique for microfluidics. An ultraviolet (UV) laser directly writes on the photoresist without a photomask, which is suitable for master fabrication. By eliminating the constraints of fixed patterns in the traditional photomask when the masters are made, this prototyping technique gives designers/researchers the convenience to revise or modify their designs iteratively. A device fabricated by this method is tested for particle separation and demonstrates good properties. This technique provides a flexible and rapid solution to fabricating microfluidic devices for non-professionals at relatively low cost.

## 1. Introduction

The last two decades have witnessed a fast development in microfluidics [1,2]. Many multidisciplinary applications found in our daily life and in the research community [3,4,5] are based on microfluidics, such as chemical analysis [3,6], biotechnology [7,8], molecular diagnostics [9,10], drug delivery and medical detection [11], microfluidic stretchable electronics [12,13,14] and soft robotics [15,16]. As an innovative technology, microfluidics provides wide-ranging tools and versatile services for research communities in chemistry [3], medicine and biology [8]. Various fabrication techniques have been developed to satisfy specific demands in different applications [17,18,19,20,21,22,23,24,25,26,27,28]. The improvement of design flexibility and rapid prototyping are concerns for microfluidic device fabrication due to frequent design modification and verification [17,18].

Among these techniques, the soft lithography based on poly(dimethylsiloxane) (PDMS) that adopted the master fabrication [17], such as photolithography and elastomer replication, for example PDMS replica [29,30], is widely employed in microfluidics. The master used in photolithography is fabricated by exposing a thick photoresist on a substrate through a photomask and the PDMS casting can replicate the precise structure from the master after PDMS curing. The soft lithography overcomes some limitations of photolithography and provides a relatively convenient, inexpensive and rapid method to fabricate microstructures for the lab-on-a-chip devices [31]. The relatively inexpensive PDMS replaces silicon as the main material in microfluidics because of its advantages in optical transparency, biocompatibility, easy moulding and flexibility [30].

The master can be obtained by photolithography without cleanroom facilities, and the photoresist processing of SU-8 for low-cost microstructure fabrication is optimized [32]. The photomask fabrication is an essential step in photolithography. Other techniques, such as deep reactive-ion etching (DRIE) [33,34], Lithographie, Galvanoformung, Abformung (LIGA) [35,36,37], and dry film photoresist [38,39] also require photomasks. These techniques involving photomask fabrication are widely adapted to fabricate masters that can achieve high resolution. The resolution of the photomask contributes to the resolution of the master, and the high-resolution photomask generator requires an advanced cleanroom environment and specialist operators. Central laboratories or providers can supply the service of photomask generation for those groups without a photomask generator. However, the additional time for photomask generation including ordering photomasks and delivery is several times longer than the time taken by mask generation itself. In addition, iterative design modification and refabrication are time-consuming.

Direct writing is an emerging micro-nano fabrication technique. Using different direct writing instruments, the structure is directly fabricated on the substrates without the photomask fabrication—for example, the xurography [21,40,41], office laser printer approach [42], hot embossed plastic microfluidic device [43], mechanical micromilling [44,45,46], laser printing on flexible copper printed circuit board (PCB) substrates [19], CO_2_ laser on poly(methyl methacrylate) [22,47] and glass [48,49]. These techniques significantly reduce the process steps in photomask fabrication and thus flexibility is enhanced. In general, the cutting plotter with a thin blade can generate the 100-µm width structure [21], and 50-µm width structure in some particular conditions [41], but the limited channel depth and low repeat accuracy cannot cover the requirements of many microfluidics. The office laser printer can generate 100-µm width lines in general, but the edge is coarse [19,42]. The hot embossed plastic device is suitable for mass production, but the accuracy is very dependent on quality of the master and the plastic. Mechanical micromilling requires a milling machine with a high-precise computer numerical controlled (CNC) system and corresponding tool library. The CO_2_ laser is a developed tool for fabrication, but it will generate thermal deformation or thermal strain, which will induce rough surface topography of the bottom channel as well as the non-vertical side channel [47]. The maskless photolithography including laser direct writing [50,51,52,53] and micromirror arrays [54,55,56] can reach a high resolution by employing the advanced instruments, but the setup cost for the instruments is beyond the affordability of most research groups. As laser fabrication technology with other direct writing technology develops, there are more choices to fabricate devices for different microfluidics [57,58]. We provide an alternative solution for master fabrication by using an ultraviolet (UV) laser direct writing on photoresist without photomask fabrication whose resolution is acceptable for most microfluidics. A device fabricated by this method is tested for particle separation and demonstrates good properties. This solution, which removes the mask fabrication process, has comprehensive advantages including time and cost savings, accuracy and robustness. These advantages are suitable for subsequence microfluidic prototyping by cutting down the time and cost between the first design and modification. Additionally, as this method is easily grasped by non-professionals in non-cleanroom laboratories, it provides a rapid and flexible prototyping technique for more people involved in microfluidics. Thus, it is highly recommended for those groups or companies who need frequent design modification and are puzzled by cost control and quality guarantee during prototyping production.

**Figure 1:**
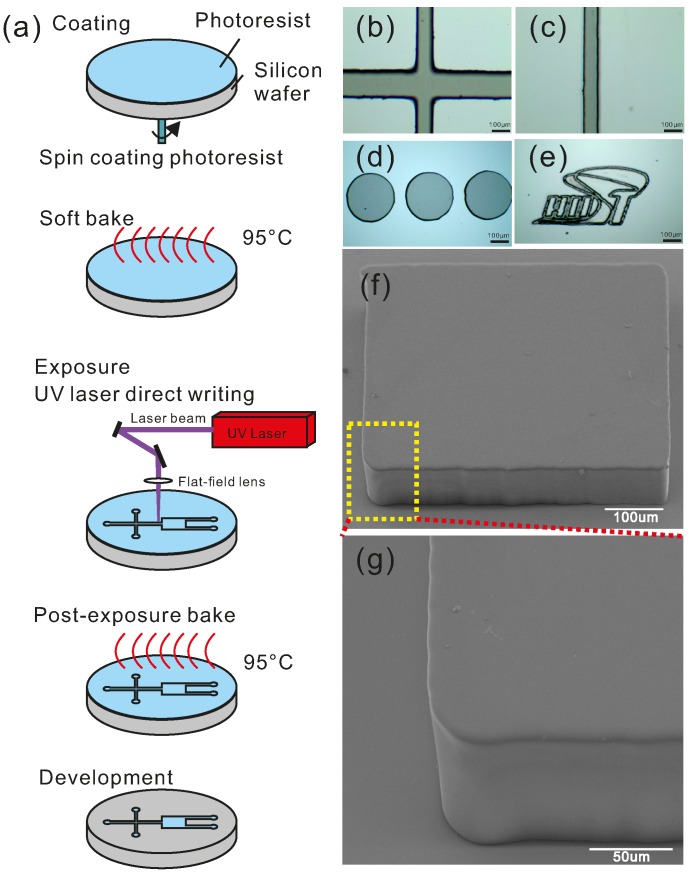
(**a**) schematic process of master fabrication; (**b**–**e**) top view images of the master structure by optical microscope; and (**f**,**g**) side view images of the master structure by scanning electron microscopy (SEM).

This paper introduces a rapid prototyping technique with high robustness and flexibility for microfluidic devices in which the soft lithography and the laser direct writing are adopted (Figure 1). This prototyping technique provides an alternative option to generate microfluidic chips for microfluidic research within 8 h. A commercial UV laser marker with a galvanometer mirror scanner is employed for laser direct writing as a substitution for the mask generator and the mask aligner. A UV laser with a wavelength of 355 nm that is close to the I-line (365 nm) in the spectrum of a UV lamp can trigger a photochemical reaction during exposure. With a standard photoresist processing protocol, a highly reliable master that is compatible with the soft lithography is obtained. To illustrate the aspects of this technique, the performance of the UV laser marker under different conditions is discussed. This method relies less on stringent requirements for equipment and professional staff. The resolution and shape-restoring ability of the structure on a master fabricated by this technique demonstrates the capacity of this technique. Finally, microparticle separation was verified for applicability with the newly developed technique.

## 2. Materials and Methods

A negative photoresist (SU-8 3025, MicroChem, Westborough, MA, USA) was uniformly spin-coated by a spin coater (WS-650, Laurell, North Wales, PA, USA) on the silicon wafer to the designed thickness. Then, the wafer with the photoresist was heated on a hot plate (EH20B, LabTech, Beijing, China) at 95 °C. At the same time, the designed patterns were sent to the UV laser marker (HGL-LSU3/5EI, Huagong Laser, Wuhan, China) and the operating parameters were also set. The silicon wafer with the photoresist was mounted on the working stage of the UV laser marker after the wafer had cooled down. The silicon wafer was then exposed to the UV laser according to the designed pattern, and placed on the hotplate at 95 °C for subsequent post-exposure baking. After baking, the silicon wafer with photoresist was immersed in the developer solution (MicroChem, Westborough, MA, USA) and the patterns emerged on the wafer. Finally, the silicon wafer was placed in an oven (DHG-9023A, BluePard, Shanghai, China) at 150 °C for 15 min for hard baking, and a robust master for microfluidics was achieved.

To ease the peeling process of PDMS from the SU-8 master, the master was coated with Trichloro(octyl)silane (Sigma-Aldrich, St. Louis, MO, USA) in a glass desiccator for 4 h before the first use. A modified soft lithography was adopted to fabricate the microfluidic chips [20]. PDMS (Elastrosil RT 601, Wacker Chemie, Munich, Germany) was mixed with a 9:1 weight ratio of base and cross-linker and stirred to form a uniform prepolymer. Then, the mixture was poured on the master and cured at 75 °C for 20 min. After the PDMS was peeled off from the master and holes were punched on the PDMS, the PDMS slabs and microscope glass slides were treated under a corona discharger (BD-50E, Energy Transfer Partners, Dallas, TX, USA) by being manually scanned back and forth under the discharging wire for 1 min, and the high-voltage treatment can cause the surface of PDMS to become hydrophilic. The treated PDMS slabs and microscope glass slides were pressed together to bond in an oven at 75 °C for 20 min.

The top views and side views of structures on the master were acquired by using a laser scanning confocal microscope (VK-X200K, Keyence, Osaka, Japan) and an scanning electron microscopy (SEM, VEGA3, Tescan, Brno, Czech), respectively. Before the SEM imaging, the master was cut into small samples (1 cm × 1 cm) by a diamond knife, and the samples were mounted on a sample holder. After the samples had been coated with a thin layer (5 nm) of gold by sputtering (Q150RS, Quorum, East Sussex, UK), the sample holder was mounted on the sample stage of the SEM. A self-developed program written in Mathematica 10.2 (Wolfram Research, Champaign, IL, USA) was used to obtain the width and roughness of the patterns, as well as the distribution of the light density.

Soft inertial microfluidics for high throughput separation has been demonstrated [59] with the newly developed fabrication technique. In such a device, four areas are included: the inlets, the focusing area, the separation area, and the outlet. The width of the channel in the focusing area is 50 μm, while the width of the channel in the separation area is 850 μm. The microfluidic chip filled with 1.5% hydroxypropyl cellulose (HPC) dissolved in 2-(4-morpholino)ethanesulfonic acid/tris(hydroxymethyl)aminomethane (MES/TRIS) buffer was kept in a closed humid container and put in the refrigerator at 4 °C overnight. The sample flow was introduced into the central inlet and the sheath flows were introduced into the upper inlet and down inlet through polyethylene (PE) tubes (PE20, Becton Dickinson, Franklin Lakes, NJ, USA), respectively. In the sample flow, the Pluronic^®^ F-127 (Sigma-Aldrich) was dissolved in deionized water to 10% solution, and then 10% bovine serum albumin (BSA) and the 10% F-127 diluted solution were added in the suspensions to prevent aggregation of the fluorescent particles (Fluoro-Max, Thermo Fisher Scientific, Waltham, MA, USA). Three syringes (Jingta, Shanghai, China) with fluidics (sample flow and sheath flows) were placed on three infusion syringe pumps (LSP02-1B, LongerPump, Baoding, China), respectively. The device was observed on the inverted fluorescence microscope. A blue-green fluorescent filter cube (Nikon B-2A, Nikon, Tokyo, Japan) and a 20× objective (NA 0.3) were used as attachments to the inverted fluorescence microscope (Nikon Ti-U, Nikon). The images of particle separation were acquired by a digital single lens reflex camera (Canon EOS 70D, Canon Inc., Tokyo, Japan) attached to the microscope, and the same program written in Mathematica 10.2 mentioned above was used to analyze the particle separation performance.

## 3. Results and Discussion

### 3.1. Fabrication Capability

The performance of the UV laser marker, which is critical to the resolution of this prototyping technique, was tested under different parameters. In applications, the exposure time should also be taken into account. The lower fabricating time is a result of the faster laser scanning speed. The bias of actual width and designed width, as well as roughness of the structure and the exposure time, are critical factors for evaluating the fabrication performance. The output energy has a negligible effect on bias, roughness or exposure time due to the low exposure energy needed for the thin SU-8 structure (<100 μm) during our test, and thus has no effect on the fabrication performance. A rectangle pattern (5 mm × 30 µm) with the height of 90 µm was designed to test the bias size and the roughness of the structure fabricated by this UV laser marker with different parameters. The parameters include the laser scanning speed (ranging from 400 to 2000 mm∙s^−1^ with a step length of 100 mm∙s^−1^), and the repetition rate of the laser (ranging from 20 to 100 kHz with a step length of 10 kHz).

**Figure 2:**
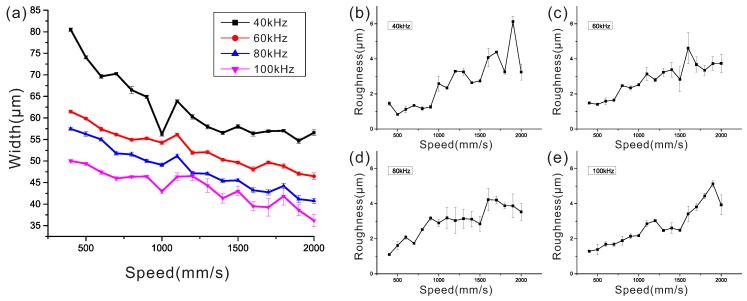
The variation tendency in width and roughness at different parameters. (**a**) the actual width of the structures versus laser scanning speed for different laser pulse repetition rates (the designed width is 30 μm); and (**b**–**e**) the measured roughness of the structures versus laser scanning speed for different laser pulse repetition rates (the designed width is 30 μm).

In Figure 2, the actual width of the structure, namely the bias of the designed width and the actual width, decreases as the laser scanning speed increases, while the roughness of the structure increases correspondingly, no matter what the repetition rate is. This finding indicates that the laser scanning speeds have a critical influence on the bias and the roughness. According to Figure 2, there is a trade-off of bias and roughness to obtain a satisfactory performance. As the laser repetition rate increases, the actual width of the structure decreases, while the roughness of the structure does not change. At a higher repetition rate, the curves of actual width and roughness look smoother and show better linear properties. Thus, the random error decreases as the repetition rate increases. A higher repetition rate results in a better fabrication resolution.

### 3.2. Bias Calibration

**Figure 3:**
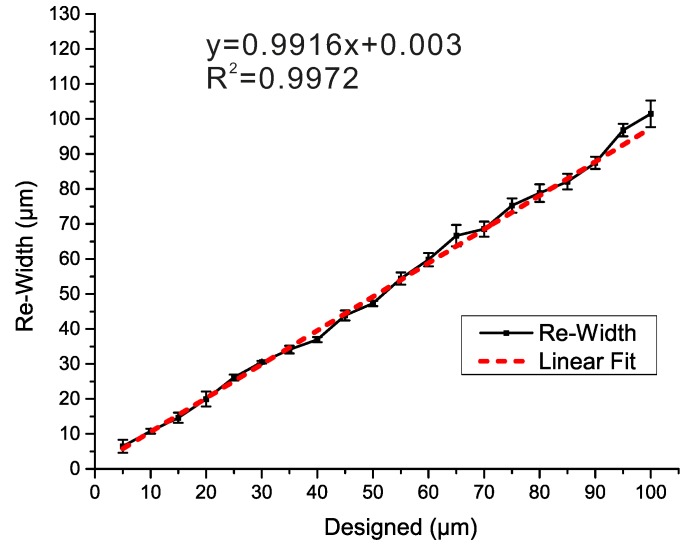
The relationship between the designed size and the actual size of the structure.

The bias between the differently designed widths and the actual widths of structure with the height of 85 µm are shown in Figure 3. The relationship between the actual size and the designed size was exploited to calibrate bias. The laser scanning speed of 1000 mm∙s^−1^ and the repetition rate of 100 kHz were chosen, as indicated above. A series of designed rectangle patterns with the same length of 5 mm and different widths (from 5 to 100 μm) were tested to acquire the actual widths and roughness of the structures. In Figure 3, the relationship of the bias between designed widths and actual widths shows good process stability of the UV laser marker. The mean width of the laser pulse is 19.621 μm, and the minimum structure width of 25 μm is achieved by this UV laser marker. The mean width of the laser pulse (19.621 μm) is subtracted from each actual width, and the remaining widths are fitted into a line (Figure 3). The depth is 85 µm, and the aspect ratio ranges from 0.7 to 3.26. Other depths ranging from 24 to 90 μm were tested, and the results show a similar linear relationship, which are not showed here. The correlation coefficient (*R*^2^) is close to 1, which shows a good linear relationship between the designed width and the actual width. In terms of width, the new master generating technique is quite suitable for most microfluidic prototyping of lab-on-a-chip devices.

**Figure 4:**
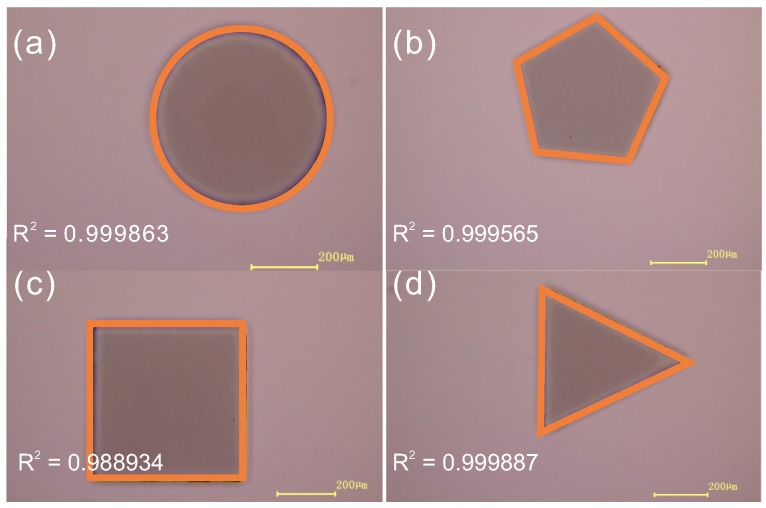
The patterns of circle (**a**); pentagons (**b**); squares (**c**); and triangles (**d**) fabricated by this technique and their correlation coefficients (*R*^2^).

Apart from the bias and roughness, the shape distortion was also tested. In microfluidics, different patterns are designed as channels. It is important to keep the shape of the pattern consistent with the designed one. In Figure 4, the patterns of circles, pentagons, squares and triangles were designed and fabricated using this technique with the height of 100 μm. The correlation coefficients of the patterns are close to 1, and all patterns show a good shape-restoring ability. In Figure 1b–d, it can be seen that this technique is good for fabricating various structures.

### 3.3. Prototyping Time Estimation and Instrumental Investment

Another important factor for prototyping fabrication is the consumed time. This prototyping technique reduces the processing time of photomask fabrication compared to soft lithography. The fabricating process can be divided into three procedures, namely idea to pattern, pattern to master and master to device. The pattern to master is the critical procedure and takes different amounts of time in the two techniques, while the idea to pattern and the master to device are the same fabricating process. The basic conditions are estimated as follows: the acreage of pattern is 1000 mm^2^; and the writing speed of a mask generator (Heidelberg DWL 66fs, Heidelberg Instruments GmbH, Heidelberg, Germany) is 10 mm^2^∙min^−1^, while the laser scanning speed of this UV laser marker is 1000 mm∙s^−1^ (that is 120 mm^2^∙min^−1^ at the filling space of 0.002 mm).

In soft lithography, the pattern to master can be divided into two steps: mask fabrication and master fabrication. For a mask-generation-equipped laboratory, the fabricating time of photomasks is about 155 min (Table 1). For no mask-generation-equipped laboratory, the delivery time will take more than 24 h. The master is fabricated by standard photolithography (Table 2). The estimated fabricating time of pattern to master by our method is listed in Table 2. For photolithography, the photoresist with the thickness of 50 µm is usually exposed four times at 10 s per time, and the waiting time between two times is 30 s. For laser scanning, it theoretically takes about 8 min to scan 1000 mm^2^ at a scanning speed of 2 mm^2^·s^−1^. Thus, the exposure times for these two methods do not have an obvious difference compared to the whole processing time. The parameters of the machine can be set during the cooling time, so the setting time is not counted in the total time.

In Table 3, the fabrication time of the PDMS device is estimated. In Table 4, the time of pattern-to-master is halved by this technique compared to soft lithography. The total process can be finished within 8 h, so it only takes one working day to fabricate a microfluidic prototype. If the laboratory is not equipped with a mask generator, communication with providers and delivery would take a significant amount of time. Our technique simplifies the process of prototyping fabrication and saves the cost of a mask generator, and pattern-to-master can be achieved in only one step by one machine.

**Figure 5:**
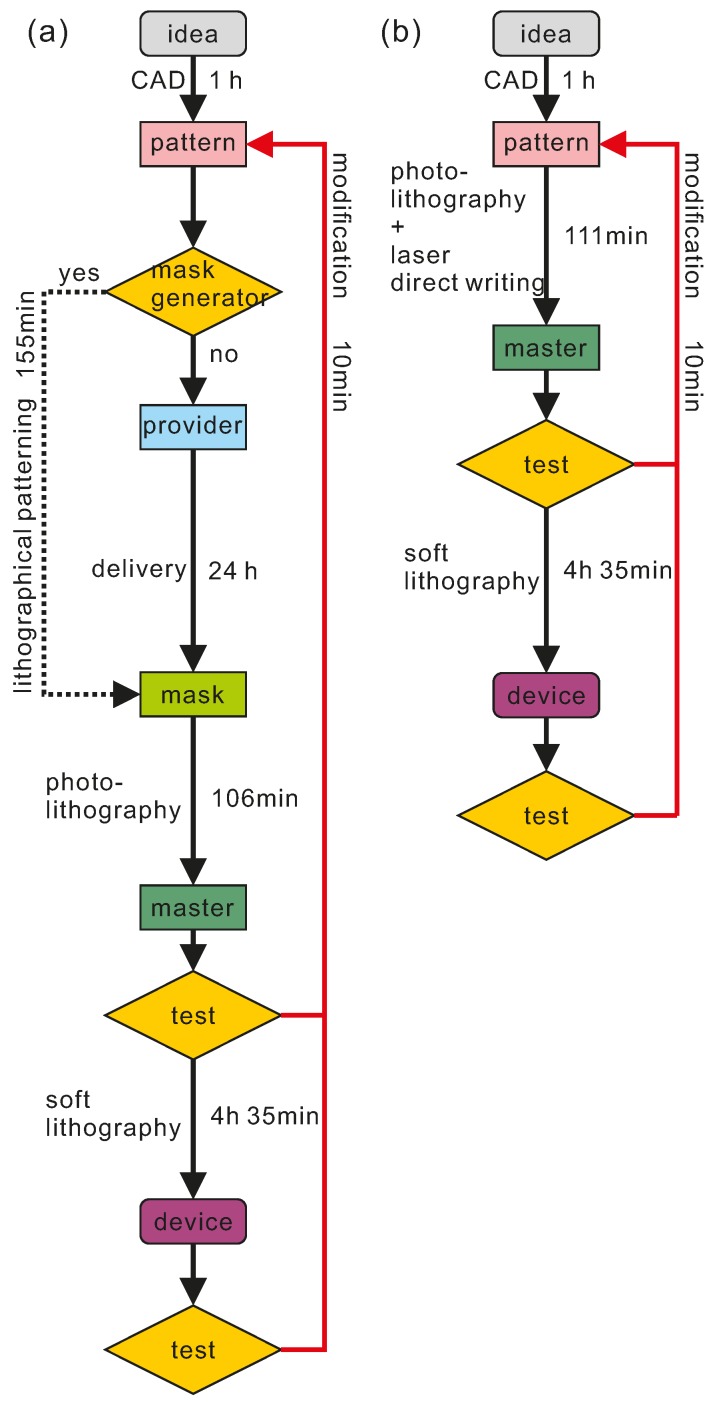
The process comparison of soft lithography with this technique. (**a**) Soft lithography; and (**b**) this prototyping technique.

The prototyping of lab-on-a-chip devices in microfluidic research always needs continuous modifications. The master and device need to be checked and the pattern modified to ensure satisfactory prototyping. By simplifying the process step of photomask fabrication, this technique provides a rapid and flexible way to modify the design and refabricate the master. To clearly compare the difference, we made a flow chart of the typical process as seen in Figure 5. We can see that this technique simplifies the process steps and saves the time of photomask fabrication significantly. The total times used from idea to pattern, master fabrication and device fabrication can be achieved in a working day. When modifications are required, the technique can save more time compared to traditional photolithography.

The main instrument in this technique is the UV laser marker, which costs $35,000. In traditional photolithography, the two main instruments are the mask generator and the mask aligner, which cost $350,000–$500,000 and $100,000–$200,000, respectively. The UV laser marker is much cheaper than both the mask generator and the mask aligner. This makes this technique affordable for many research groups.

Compared to other prototyping techniques (Table 5), this method provides a solution for microfluidic prototyping that has compromises in time, cost, flexibility and accuracy.

### 3.4. Microfluidic Application Demonstration

**Figure 6:**
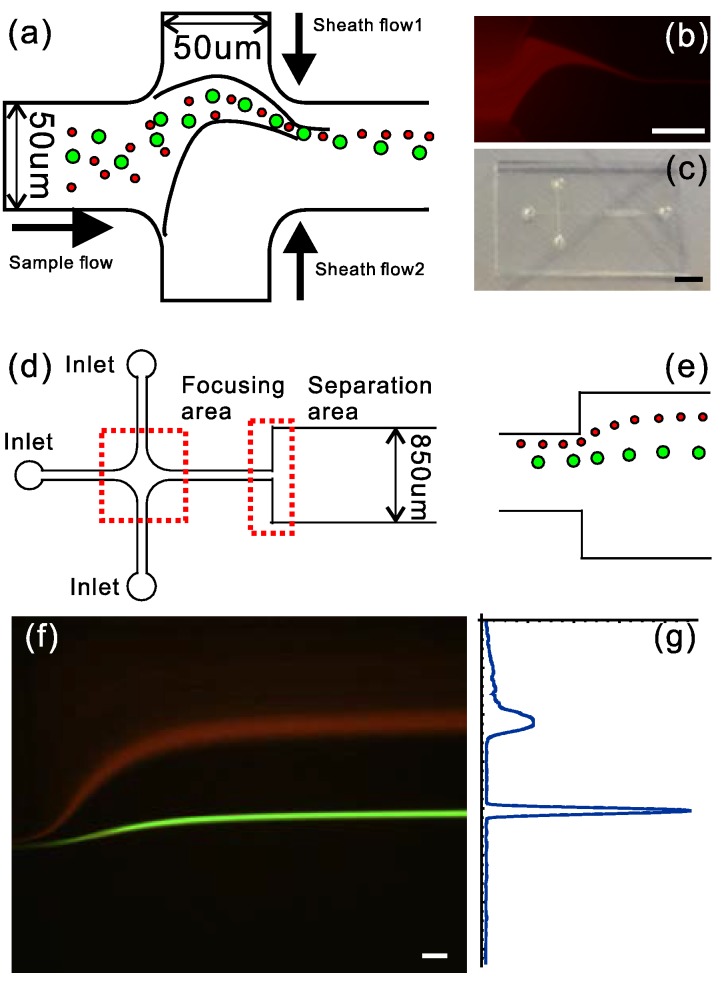
Application of this microfluidic prototyping fabrication for separation. (**a**) schematic diagram of the focusing area by asymmetric sheath flows (the flow rate of sample flow, sheath flow 1 and sheath flow 2 are 2, 9 and 45 µL·min^−1^, respectively); (**b**) the asymmetric focusing area through the invent fluorescent microscope (the scale bar is 50 μm); (**c**) the image of the microfluidic chip (the scale bar is 4 mm); (**d**) the design of the microfluidic chip for separation; (**e**) the schematic diagram of the separation area; (**f**) the image of the separation of big microparticles (green, Φ 9.9 μm) and small microparticles (red, Φ 1.0 μm) through the invented fluorescent microscope (the scale bar is 50 μm); and (**g**) the distribution of the particles according to the intensity of light.

The sample flow carrying the microparticles is introduced into the middle inlet and is focused to form a thin, curved stream by the asymmetric sheath flows [59]. The flow rate of sheath flow 2 (*Q*_2_) is much higher than the sample flow rate (*Q*), and it can protect one side of the channel wall. The flow rate of sheath flow 1 (*Q*_1_) is lower than the sample flow rate to protect the other side. In the focusing area, the large particles suffer more inertial force and are deflected away from the original carrier flow while the small particles can follow the original carrier flow. The large particles and small particles separately follow different trajectories. The separation distance is amplified in the separation area. The soft inertial force introduced by asymmetric sheath flow has been utilized in microfluidics for separation (Figure 6a). The minimum width of the channel is 50 μm, and the thickness of the channel is 38 μm while the height of the channel is 55 μm. The flow rates of sample flow, sheath flow 1 and sheath flow 2 are 2, 9, and 45 μL∙min^−1^, respectively. The sample flow was focused to form a thin, curved stream by asymmetric sheath flow rates in the focusing area (Figure 6b). The small particles tend to flow along the streamline while the large particles tend to flow away from the streamline due to the higher inertial force. In the separation area, the large particles flow in the central channel and the small particles flow near the upper wall. The microfluidic chip was fabricated by this technique (Figure 6c,d) and the large particles and small particles were separated in the separation area (Figure 6e). The separation of big particles (Φ 9.9 µm, the green line) and small particles (Φ 1.0 µm, the red line) was achieved as shown in Figure 6f. The distribution of light intensity of Figure 6f is shown in Figure 6g.

## 4. Conclusions

A flexible and rapid prototyping technique was presented for microfluidic devices by employing a UV laser marker. A resolution of 25 μm has been achieved and we demonstrate a microfluidic device for separation. Removing the procedure of fabricating a mask saves on costs and time of microfluidic prototyping and simplifies the process of photolithography, particularly when additional time for modifications is needed. The flexibility and resolution of this technique make it well suitable for microfluidic prototyping in the research community.

Although only SU-8 photoresist was tested by this method, this method is expected to utilize different photoresists, including positive photoresists, to fabricate different kinds of microfluidic chips for separation, micromixing, droplet formation, cell culture, drug delivery, point-of-care and more wide-ranging applications. The resolution of this technique can be further enhanced by improving performance of the laser and the optical system required.

## Figures and Tables

**Table 1:** The estimated time of fabricating the photomask.

Process Steps	Time (min)
Enter cleanroom	10
Lithographic patterning ^1^	115
Development	2
Rinsing and drying	5
Etching	10
Photoresist removing	5
Mask drying	3
Total	155

^1^ This process includes setting the parameters of the machine (10 min) and placing the pre-coated mask (5 min) and laser writing (100 min).

**Table 2:** The estimated time of fabricating the master.

Process Steps	Photolithography (min)	Our Method (min)
Wafer cleaning	30	30
Dehydration bake (200 °C)	5	5
Cooling time	5	5
Spin-coating photoresist (SU-8)	2	2
Soft bake	20	20
Cooling time	10	10
Exposure	3	8
Post-exposure bake	6	6
Development	10	10
Hard bake	15	15
Total	106	111

**Table 3:** The estimated time of fabricating the poly(dimethylsiloxane) (PDMS) device.

Process Steps	Time
Coating master ^1^	4 h
Mixing PDMS ^2^	5 min
Degassing ^3^	20 min
Curing	20 min
Punching hole and cleaning	5 min
Bonding	10 min
Total	4 h 35 min

^1^ The new master should be coated before use. It is not necessary to coat it again for the next time; ^2,3^ These two steps can be implemented during the master coating process, thus the time taken is not counted in the total time.

**Table 4:** The comparison in fabrication time between soft lithography and our method.

Method	Idea to Pattern	Pattern to Master	Master to Device	Total
Soft lithography	1 h	Mask fabrication: 155 min	4 h 35 min	596 min
		Master fabrication: 106 min		
Our method	1 h	111 min	4 h 35 min	446 min

**Table 5:** The comparison of this method with other prototyping techniques.

Method	Resolution	Roughly Estimated Prototyping Time (Dependence on Pattern)	Main Instruments	Advantages	Disadvantages
This method	25 µm	3 h	UV laser machine	Flexible, good accuracy	Planar structure
Dry photoresist [40]	20 µm	2 h	UV oven	Fast, convenience	Mask-based necessary
Xurography [21]	100 µm	1.5 h	Cutting plotter	Fast, convenience	Limited minimum dimension and materials
Laser printer [43]	50 µm	3 h	Laser printer	Fast, convenience	Rough edge
Micromilling [45]	30 µm	4 h	Micromilling machine	Semicircular channel, durable master	High-cost initialization and easily tool breakage
CO_2_ laser [22]	50 µm	3 h	CO_2_ laser machine	Fast, convenience	Rough edge and bottom
Maskless photolithography [51]	1.5 µm	6 h	Direct laser writing machine	High precision	High-cost initialization
